# Influence of healthy lifestyle behaviors on life satisfaction in the aging population of Thailand: a national population-based survey

**DOI:** 10.1186/s12889-020-10032-9

**Published:** 2021-01-06

**Authors:** Sirinya Phulkerd, Sasinee Thapsuwan, Aphichat Chamratrithirong, Rossarin Soottipong Gray

**Affiliations:** Institute for Population and Social Research, Mahidol University, Nakhon Pathom, 73170 Thailand

**Keywords:** Life satisfaction, Lifestyle behaviors, Older persons, Well-being, Aging

## Abstract

**Background:**

Understanding the influence of healthy lifestyle behaviors on population-level life satisfaction is few known in the aging population, especially in low- and middle-income countries in Asia. The objective of our study was to analyse the association of lifestyle behaviors with life satisfaction in a nationally-representative sample of older persons in Thailand.

**Methods:**

The sample was obtained from a baseline phase of a nationally-representative, longitudinal survey of the Thai population. The study employed a multistage sampling technique to recruit study participants age 60 years or older from the five geographic regions of Thailand. In this study, 1460 adults age 60 years or older from 3670 households successfully completed face-to-face interviews by trained staff with a structured questionnaire. Information on self-reported life satisfaction, lifestyle behaviors, and sociodemographic characteristics were collected via survey questionnaire. Life satisfaction was assessed using the Scale with Life Satisfaction (SWLS) [1 to 7] response. Binary logistic regression analysis was used in investigating the association between lifestyle behaviors and life satisfaction.

**Results:**

The median age of the participants was 68.1 (60–93 years). The overall mean life satisfaction score was 24.2 ± 5.6. Regular physical activity (at least 30 min per day) and sufficient fruit and vegetable (FV) intake (at least 400 g per day) were significantly associated with older people’s life satisfaction (*p* ≤ 0.001 and *p* ≤ 0.10, respectively) after controlling all sociodemographic variables. Participants who had regular physical activity were 1.7 times as likely to be satisfied as those with less physical activity (95% CI 1.284–2.151). Participants with sufficient daily FV intake were 1.3 times as likely to be satisfied with life as those with insufficient daily FV (95% CI 0.994–1.723). Life satisfaction score also differed significantly by sociodemographic characteristics (sex, age, marital status, educational attainment) and presence of chronic disease.

**Conclusions:**

To improve the life satisfaction of older persons, taking into account sociodemographic characteristics of the population and absence of chronic disease, the need for promotion of healthy lifestyle behaviors, especially regular physical activity and sufficient FV intake, must be recommended.

**Supplementary Information:**

The online version contains supplementary material available at 10.1186/s12889-020-10032-9.

## Background

The impact of healthy lifestyle behaviors on health is well known, in particular the association between lifestyle behaviors and well-being. The changes in lifestyle (such as diet, exercise, smoking, and alcohol) are regarded as indirect mediating links between health-related behaviors and psychological factors such as life satisfaction [1, 2]. Life satisfaction is a subjective proxy measure of personal well-being which can contribute to physical and mental health, as well as complement more objective indicators. Life satisfaction is grounded in people’s preferences rather than in a-priori judgments about what are the important drivers of an individual’s well-being [3].

Relationships between diet and life satisfaction in aging populations were demonstrated [4–7]. Older persons with healthy diets had higher life satisfaction than those with unhealthy diets [4]. Life satisfaction was associated with higher contribution of organic food among Finnish participants over 45 years old [6]. In South Korea, the effects of degree of nutritional diet on life satisfaction in older persons was showed [7]. The usual physical activity was positively associated with life satisfaction in adulthood in particular middle and older adults [8]. Current smoking and excess alcohol consumption affected one or more domains of life satisfaction (overall life satisfaction, psychological health satisfaction, and/or somatic health satisfaction) [9]. In turn, the life satisfaction of smokers who would like to quit smoking, increase when smoking bans were implemented [10]. Low life satisfaction score influenced coping expectation, and therefore increased alcohol consumption [11].

More concepts explain possible causal mechanisms of action linking fruit and vegetable (FV) intake and psychological health. The majority of the concepts are centered on the ability of certain nutrients contained within FV to influence psychological well-being. Some promising nutrients such as complex carbohydrates (found in certain types of FVs e.g. beans, peas and bananas), and Vitamins B, C and E were found in relation to psychological functioning, particularly mood. Carbohydrates trigger insulin influences a range of functions from memory to the regulation of mood [12]. B vitamins have beneficial effects on reducing stress, mild psychiatric symptoms and anxiety [13]. Antioxidant properties of vitamins C and E have a protective effect against oxidative stress [14]. Certain minerals contained within FV e.g. iron [15], calcium and magnesium [16] are also linked to improvements in psychological health such as reducing depression and stress.

Despite a growing interest in investigating life satisfaction of aging populations and its association with lifestyle behaviors, only a few studies have focused on older Asian adults, especially in low- and middle-income countries which are progressing from aged to super-aged such as Thailand. Previous studies highlighted the importance of healthy lifestyle behaviors in increasing life satisfaction among aging populations [1, 4, 5, 8, 9, 17]. For policymakers, an examination of life satisfaction and associated lifestyle-related factors is recommended. This information may provide important implications for developing public health strategies to promote and maintain good health and well-being of older persons in later life. According to the active aging approach promoted by the World Health Organization, adopting healthy living practices throughout life permits to maintain good health beyond age 60, including physical, social, and mental well-being [18].

The aims of our study were to measure life satisfaction score and to analyse the association of lifestyle behaviors with life satisfaction score in older persons in Thailand after controlling for sociodemographic characteristics, using nationally-representative survey data.

## Methods

### Data collection and study population

The sample of this study was obtained from the first wave (baseline phase) of the Fruit and Vegetable Eating Behaviors Study which is a nationally-representative, longitudinal survey of the Thai population. The study employed a multistage sampling technique to recruit study participants from the five geographic regions of Thailand (Bangkok, Central, North, Northeast and South regions). Although Bangkok’s boundaries are at the provincial level, unlike the other provinces, it is a special administrative area with its own legislative body, power and municipal budget, and has regional-level sociodemographic characteristics, especially urbanization and political economy. Accordingly, the Thai National Statistical Office (NSO) usually classifies Bangkok as a separate area from other regions, and classifies it as an urban area when conducting nationwide surveys.

Details of the sampling technique are described in another study [19]. Briefly, within each geographic region two provinces were randomly selected and then, within each province, enumeration areas were sampled. The ultimate sampling units were the households, and all individuals who resided in the selected household and were present during data collection were interviewed. The survey included a representative sample of individuals age 60 years or above in Thailand. The data in the study was obtained from May to December 2018, which includes data on various health-related topics and also sociodemographic characteristics. In this study, 3720 households were randomly sampled, within which 3670 successfully completed the survey. An overall response rate of 98.7% was achieved. A total of 1460 adults age 60 years or older successfully completed face-to-face interviews by trained staff with a structured questionnaire.

### Measures

Details of the measures used in this study are briefly described below.

#### Life satisfaction levels

Life satisfaction score was used as the outcome variable to measure the level of life satisfaction among Thai older persons. A participant’s level of life satisfaction was assessed using the 1–7 response scale derived from the Scale with Life Satisfaction (SWLS) instrument, invented by Diener and colleagues [20]. The SWLS has been used widely and has proven as a valid and reliable measure of the life satisfaction component of subjective well-being in diverse population groups. The SWLS has been translated in many different languages including Thai (available at *https://eddiener.com/scales/7**).*

The participants were told to indicate their level of agreement with each of five statements below using the 1–7 scale: ‘7 - Strongly agree,’ ‘6 - Agree,’ ‘5 - Slightly agree,’ ‘4 - Neither agree nor disagree,’ ‘3 - Slightly disagree,’ ‘2 - Disagree,’ ‘1 - Strongly disagree.’

‘In most ways my life is close to my ideal.’

‘The conditions of my life are excellent.’

‘I am satisfied with my life.’

‘So far I have gotten the important things I want in life.’

‘If I could live my life over, I would change almost nothing.’

A higher score indicates a higher level of satisfaction. A high internal consistency of the scale was anticipated. This scale shows an acceptable reliability in this study (Cronbach’s alpha was 0.738). A one-dimensional structure was confirmed by a factor analysis.

#### Sociodemographic variables

Age, sex, marital status, place of residence and educational attainment were self-reported by the participants. Age was categorized into three groups: ‘60–69,’ ‘70–79,’ and ‘80 or above,’ to distinguish between life stages which are youngest-old, middle-old and oldest-old according to the Thai NSO [21]. Sex was included as a dichotomous variable; i.e., male or female. Marital status was classified into three groups: ‘single,’ ‘married,’ and ‘widowed/divorced/separated.’ Place of residence was divided into ‘urban area (including Bangkok)’ and ‘rural area.’ Educational attainment was classified into ‘no formal education,’ ‘primary education,’ ‘secondary education’ and ‘bachelor’s degree or higher.’

#### Lifestyle behavior and health-related variables

Smoking and alcohol histories were included in the analysis and were categorized into three groups: ‘never-smoker/drinker,’ ‘ex-smoker/drinker,’ and ‘current smoker/drinker’ (including ‘occasional smoker/drinker’ and ‘daily smoker/drinker’). Daily physical activity at moderate or vigorous level such as brisk walking, running, aerobics, and competitive games or sports for at least 30 min per day was recorded as ‘yes’ and ‘no.’

FV consumption of the participants was also collected. The FV consumption was recorded based on the frequency (number of days) that the participant consumed FV in a typical week, and quantity (number of servings) consumed on each day. Servings were determined based on response after showing pictures of raw and cooked FV items in one serving size, adapted from the ‘Healthy Eating Guidelines’ from the Bureau of Nutrition, Department of Health, Ministry of Public Health, Thailand [22]. Details of the standard serving for FV are described in another study [19]. The FV consumption combined was categorized into two groups according to recommendations by the World Health Organization (WHO) (at least five servings or 400 g per day) [23]: ‘less than 400 grams,’ and ‘400 grams or above.’

Other health-related variables were health status and denture wearing. Health status was defined as the presence of a physical chronic disease such as cancer, diabetes, hypertension, stroke, or asthma. Participants were asked whether they have any chronic disease(s) and, if so, what those chronic disease(s) are. The participants were asked to recall if they have any chronic condition or disease that require ongoing medical attention (as diagnosed and prescribed by a doctor or other health personnel such as medical officer, nurse, or public health worker). Denture wearing was positively hypothesized to be related to oral-health-related quality of life (OHRQoL) [24], which has a significant association with frequency of fruit and/or vegetable consumption among older persons [25]. In this study participants were asked whether they wear dentures. They were classified as wearing dentures or not. The questionnaire used in this study is shown in Supplementary File 1.

### Data analysis

Descriptive statistics were used in describing level of life satisfaction, and examining the association between life satisfaction and each of the independent variables, using analysis of variance and the χ2 test. The mean and standard deviation (SD) were calculated.

The main hypothesis is that older persons who have a healthy lifestyle will be more satisfied with their lives than those having an unhealthy lifestyle. A corollary hypothesis is that people’s life satisfaction differs by sociodemographic characteristics and other health-related factors.

The likelihood of being satisfied with one’s life was determined by binary logistic regression analysis. Binary regression analysis was used to examine the association between each independent variable with life satisfaction score. Odds ratios (ORs) were calculated and estimates were presented with a 95% confidence interval (CI). The OR was used as a measure of association between the lifestyle behavior variables and life satisfaction score. Any observed relationship with a *p* value of 0.05 or less (2-tailed) was considered statistically significant.

The dependent variable of the analysis was life satisfaction score, operationalized as a binary response variable. After calculating participants’ overall life satisfaction score, participants were classified into two groups – participants with ‘less life satisfaction’ (those who have overall life satisfaction scores between 5 and 20 = 0) and participants with ‘more life satisfaction’ (those who have overall life satisfaction scores between 21 and 35 = 1). The rationale of using cutoff scores at 20 was according to Pavot and Diener (2008), indicating a score of 20 representing the neutral point on the SWLS.

Independent variables in the logistic regression equation were age, sex, marital status, place of residence, educational attainment, denture wearing, smoking, alcohol drinking, regular physical activity, FV intake, and presence of chronic disease. These variables were included in the model based on previous literature that showed an association with life satisfaction or psychosocial well-being [1, 4, 5, 8, 9, 17, 26, 27].

In the analysis, the reference groups for the variables are as follows: male for gender, 60–69 years for age, being single for marital status, living in a rural area for place of residence, no formal education for education, not wearing dentures for denture, current smoker for smoking, current alcohol drinker for alcohol, irregular physical activity for physical activity, less than 400 g per day for FV intake, and currently having more than two chronic diseases for presence of chronic disease/condition.

## Results

### Demographic and health characteristics of the sample

Of the 1460 respondents, 656 (44.9%) were men and 804 (55.1%) were women; the median age of the participants was 68.1 (ranging from 60 to 93 years). Over two-thirds of the participants were married (69.1%), and 80.2% of the participants had primary education. Almost three-fourths of participants did not wear dentures. About two-thirds of the participants lived with at least one chronic disease (67.6%).

### Life satisfaction levels

Life satisfaction scores were divided into two groups: 1) less satisfaction [5–19]; and 2) more satisfaction [20–34]. The majority of the participants (75.7%) reported having more satisfaction with life (Table 1).

**Table 1:** Overall life satisfaction of Thai elderly population

Life Satisfaction levels	N	%
Less life satisfaction (scores 5–20)	355	24.3
More life satisfaction (scores 21–35)	1105	75.7
**Total**	**1460**	**100.0**

Table 2 shows the distribution of overall life satisfaction in the study population. The average life satisfaction score among the participants was 24.2 (SD = 5.61). Statistically significant differences were found in relation to age, alcohol drinking, physical activity and FV intake.

**Table 2:** Life satisfaction in Thai older persons according to the sociodemographic and health-related factors (median, mean and standard deviation (SD))

Variables	N	% of total	Median	Life satisfaction		
				Mean	SD	***P***-value
**Total**	**1460**	**100.0**	**25.0**	**24.2**	**5.611**	**–**
**Sex**						
Male	656	44.9	25.0	24.1	5.684	
Female	804	55.1	25.0	24.3	5.552	0.656
**Age (years)**						
60–69	919	62.9	25.0	23.8	5.928	
70–79	446	30.6	26.0	24.9	4.940	**0.000**
80 or over	95	6.5	26.0	24.9	5.044	0.067
**Marital status**						
Single	59	4.1	22.0	23.2	5.036	
Married	1009	69.1	25.0	24.2	5.594	0.523
Widowed/divorced/separated	392	26.8	25.36	24.2	5.734	0.365
**Education attainment**						
No formal education	63	4.3	24.0	22.7	6.133	
Primary school	1171	80.2	25.0	24.1	5.563	0.144
Secondary school	173	11.8	25.0	24.5	5.631	0.291
Bachelor’s or higher degree	53	3.7	28.0	26.2	5.458	0.151
**Place of residence**						
Rural	856	58.7	25.0	24.0	5.595	
Urban	604	41.3	25.0	24.4	5.630	0.608
**Denture wearing**						
No	1088	74.5	25.0	24.0	5.735	
Yes	372	25.5	26.0	24.8	5.184	0.057
**Smoking**						
Current smoker	220	15.0	25.0	23.7	5.613	
Ex-smoker	333	22.8	25.0	24.4	5.977	0.270
Never-smoker	907	62.2	25.0	24.2	5.468	0.860
**Alcohol drinking**						
Current drinker	299	20.4	26.0	24.6	5.272	
Ex-drinker	447	30.7	25.0	23.9	6.126	**0.010**
Never-drinker	714	48.9	25.0	24.2	5.404	0.776
**Regular physical activity**						
No	775	53.1	24.09	23.5	5.873	
Yes	685	46.9	26.0	25.0	5.194	**0.000**
**FV intake (grams/day)**						
Lower than 400	990	67.8	25.0	24.0	5.799	
400 or above	470	32.2	25.0	24.5	5.185	**0.003**
**Presence of chronic disease**						
> 2 diseases	110	7.5	23.0	22.3	5.814	
2 diseases	334	22.9	25.0	24.3	5.486	0.353
1 disease	543	37.2	25.0	24.4	5.569	0.307
No	473	32.4	25.0	24.3	5.638	0.414

*P*-value from mean

Participants age 60–69 years and 70–79 years had significant differences in life satisfaction (*p* = 0.000). Current drinkers had significantly different life satisfaction compared to ex-drinkers (*p* = 0.010). People with different lifestyle behaviors in terms of regularity of physical activity (*p* = 0.000) and amount of FV intake combined (*p* = 0.003) were found to have significant differences in life satisfaction.

### Regression of life satisfaction on lifestyle behaviors and other associated factors

Table 3 shows results from the logistic regression analysis. The outcome (an OR) of each category of all variables is presented in relation to the omitted category shown as the reference group in the table and in the previous section. The binary logistic regression analysis shows that physical activity and FV intake were significantly related to life satisfaction among Thai older persons (*p* ≤ 0.001 and *p* ≤ 0.01, respectively). Participants who had regular physical activity were 1.7 times as likely to be satisfied with life as those with less physical activity (adjusted OR = 1.662, 95% CI 1.284–2.151). Participants with sufficient daily FV intake were 1.3 times as likely to be satisfied with life as those with insufficient daily FV intake (adjusted OR = 1.309, 95% CI 0.994–1.723). There was no significant association of smoking and alcohol drinking with life satisfaction.

**Table 3:** Binary logistic regression of sociodemographic characteristics, health status, and lifestyle behavior of older persons (*N* = 1460)

Variables	Unadjusted OR		Adjusted OR	
	(95% CI)	***P-***value	(95% CI)	***P-***value
**Sex (Reference group = Male)**				
Female	1.004 (0.789–1.276)	0.997	1.512 (1.034–2.212)	0.033
**Age (years) (Reference group = 60–69)**				
70–79	1.815 (1.368–2.408)	0.000	2.082 (1.543–2.808)	0.000
80 or over	1.536 (0.913–2.583)	0.106	1.886 (1.088–3.268)	0.024
**Marital status (Reference group = Single)**				
Married	2.287 (1.336–3.916)	0.003	2.554 (1.414–4.613)	0.002
Widowed/divorced/separated	2.189 (1.241–3.858)	0.007	2.152 (1.165–3.976)	0.014
**Education attainment (Reference group = No formal education)**				
Primary school	1.519 (0.883–2.616)	0.131	1.846 (1.043–3.264)	0.035
Secondary/high school	1.411 (0.753–2.643)	0.282	1.485 (0.759–2.905)	0.248
Bachelor’s or higher	3.062 (1.193–7.860)	0.020	3.918 (1.457–10.533)	0.007
**Place of residence (Reference group = Rural)**				
Urban	1.176 (0.921–1.503)	0.194	1.165 (0.900–1.509)	0.246
**Denture-wearing (Reference group = No)**				
Yes	1.264 (0.952–1.679)	0.105	1.237 (0.920–1.662)	0.159
**Smoking (Reference group = Current smoker)**				
Ex-smoker	1.045 (0.698–1.563)	0.832	1.020 (0.665–1.565)	0.927
Never-smoker	0.928 (0.657–1.311)	0.672	0.745 (0.470–1.183)	0.212
**Alcohol drinking (Reference group = Current drinker)**				
Ex-drinker	0.934 (0.662–1.316)	0.695	0.930 (0.647–1.337)	0.695
Never-drinker	0.954 (0.695–1.311)	0.772	0.949 (0.652–1.382)	0.786
**Exercise (Reference group = No)**				
Yes	1.648 (1.290–2.105)	0.000	1.662 (1.284–2.151)***	0.000
**FV intake (Reference group = Less than 400 g)**				
400 g or above	1.358 (1.042–1.770)	0.023	1.309 (0.994–1.723)	0.056
**Chronic Disease (Reference group= > 2 diseases)**				
2 diseases	1.709 (1.082–2.702)	0.022	1.546 (0.956–2.500)	0.075
1 disease	2.015 (1.304–3.115)	0.002	1.933 (1.219–3.065)	0.005
No	2.114 (1.356–3.294)	0.001	2.269 (1.411–3.650)	0.001

Cox and Snell *R*^*2*^ (for adjusted model) = 0.053, and for each independent variable (for unadjusted model): sex = 0.000, age = 0.013, marital status = 0.006, education attainment = 0.004, place of residence = 0.001, denture-wearing = 0.002, smoking = 0.000, alcohol drinking = 0.000, exercise = 0.011, FV intake = 0.004, and chronic disease = 0.008

The results also point to other factors associated with life satisfaction. These factors include sex, age, marital status, educational attainment, and presence of chronic disease. Female older persons were 1.5 times as likely to be satisfied with life as male older persons (adjusted OR = 1.512, 95% CI 1.034–2.212). The probability of being satisfied with their life among people at middle-old age (adjusted OR = 2.082; 95% CI 1.543–2.808) and oldest-old age (adjusted OR = 1.886; 95% CI 1.088–3.268) was much higher than those at youngest-old age. The participants who were married or widowed/divorced/separated were 2.6 and 2.2 times as likely to be satisfied with life, respectively. The participants who completed formal education were more likely to be satisfied with their life than those who had no education. The participants with a bachelor’s degree had the highest probability of being satisfied with life (among educational attainment groups) (adjusted OR = 3.918, 95% CI 1.457–10.533).

The study also found a significant association between the participants with no chronic disease and life satisfaction. The participants with no illness were 2.3 times as likely to be satisfied with life (adjusted OR = 2.269; 95% CI 1.411–3.650). No association was found between place of residence and denture-wearing, and life satisfaction.

## Discussion

Drawing on data from a nationally-representative sample of the aging population in Thailand, we examined lifestyle behaviors associated with life satisfaction level. This study is first investigation to measure life satisfaction score and associated factors among older persons in Thailand or in ASEAN member countries. Our finding show that a relationship exists between life satisfaction level and healthy lifestyle behaviors- in particular, physical activity and FV intake.

The overall mean life satisfaction score of the study group was 24.2, which corresponds to being more satisfied in life. This mean score was higher than in many countries using the same diagnostic (i.e., SWLS) such as Spain [28], Mexico [29], and Korea [30]. The differing results among countries may be linked to demographic and/or socio-cultural factors [31] and also methodological differences.

The main findings of this study show that regular physical activity (at least 30 min per day) was associated with life satisfaction level among Thai older persons. It is consistent with previous studies which reported that older people who participate in regular recreational activities had higher life satisfaction scores [32, 33]. Regular physical activity is recommended as an important component of healthy aging that can produce health benefits in improving mental and physical health, and functional status of older persons [34, 35]. Physical activity stimulates a release of dopamine, noradrenaline, and serotonin which can boost mood and overall well-being [36]. WHO [37] recommends that, in order to improve physical health and reduce risks of non-communicable disease (including mental illness), older persons (i.e., age 65 years or older) should engage in regular physical activity throughout the week. At least 150 min of moderate-intensity aerobic physical activity, or at least 75 min of vigorous-intensity aerobic physical activity, or an equivalent mix of moderate- and vigorous-intensity activity. This level of activity can boost beneficial brain chemicals as well as confer additional health benefits for older persons. Thus, encouraging older persons to take part in regular physical activity in order to improve life satisfaction, and as a means to improve their overall health is recommended.

Another interesting finding is that the FV consumption was linked to life satisfaction level. This was not surprising, since the prevailing literature shows that FV consumption favorably affects a person’s physical and mental condition [38–40]. A large cross-sectional study in the UK found an association between increased FV consumption and increased self-reported life satisfaction, as well as other positive mental states, even after controlling for demographic and economic factors [41]. Those findings suggest that higher FV intake may be associated with greater well-being. Similar to a longitudinal study of Australian adults, the level of FV consumption was predictive of increased life satisfaction. The more FV consumed, the higher life satisfaction people gained [42]. For the Thai government, it is important to continue to promote FV consumption among its citizens, especially the older persons because a recent survey found that 70% of elderly Thais had insufficient FV intake [19]. Interventions are needed to increase access for older persons to the appropriate amount of FV to ensure a balanced, adequate and varied FV diet to boost immunity and healthy development.

In this study, people’s life satisfaction differed by sociodemographic characteristics (sex, age, marital status, education) and other health-related factors (presence of chronic diseases). Study’s female older persons were more likely to have life satisfaction score than male older persons; this may be related to gender norms. Thai women are expected to perform the bulk of caregiving in the household or family, while also fulfilling their role as wife, partner, mother and overall household caretaker. Multiple roles means that women have more social interaction with a variety of people through social activities with their children, family members and neighbors, compared to men -- who usually play the primary breadwinner role of the family. Social interaction, especially with loved ones and close acquaintances, both formally and informally, can confer quality of life such as emotional support that promotes both mental and physical health [43].

In our findings, a positive association between age and life satisfaction level exists; this could be explained by the fact that the majority of Thai older persons (age 70 years or older) can no longer work in strenuous jobs, and are dependent on family for support and live together with the family. In 2017, the Survey of the Older Persons in Thailand [21] shows that 89.2% of older persons lived with family while 10.8% lived alone. Living with family (especially multiple generations) gives older persons access to more social support and interaction which, in turn, can buffer stress and promote well-being and quality of life. A relationship was observed between marital status and life satisfaction; married people were more likely to be satisfied with life than those who were single. Living with an elderly spouse is seen as more beneficial than living alone because a couple can provide mutual social support and care [44]. Accordingly, marriage can reinforce healthy lifestyle practices and better monitoring of each other’s health behaviors, and thus contribute to better health and well-being. It is consistent with previous results showing that long-term marriage has positive effects on a couple’s well-being, whereas marital dissolution introduces stress [45]. However, this effect of marriage depends primarily on conjugal quality, not on marital status per se [45, 46]. Therefore, strengthening social support of the family and increasing the social network of friends and community can be used as a strategic factor to help improve life satisfaction among Thai older persons.

The higher educated older persons were more likely to have better life satisfaction than their lesser-educated counterparts. This corroborates the findings of a previous study [47], and could be explained by the fact that education assists in equipping people with skills and resources which help them to adjust to changes in their socio-economic status after retirement. According to the 2017 Older Persons Survey, 81.0% of Thai older persons had their own house [21].

Another secondary finding is that a correlation is present between presence of chronic illness and life satisfaction. Having no or less chronic disease was associated with greater life satisfaction. That finding could be explained by the fact that presence of chronic disease often impairs the capacity and function of older persons. This could restrict an individual’s mobility as well as putting them at heightened risk of disability and other related complications which, in turn, tends to be negatively associated with social participation. Social interaction can encourage people to help and be helped by others, thus leading to increased life satisfaction [48]. This result may reflect the importance of organizing and strengthening the health and social care service system to meet older people’s needs for preventing and controlling chronic illnesses and other debilitating conditions. If these basic needs are not met, it will be difficult to be satisfied with one’s life.

### Strengths and limitations

First, the original survey was cross-sectional and, thus, the statistically significant relationships identified in this study are non-directional. The association between older persons’ lifestyle behaviors and life satisfaction level could be reciprocal. Moreover, all data were self-reported, and that might inevitably introduce some bias or inaccuracy in recall and reporting. Therefore, additional research using longitudinal data are required to examine the reciprocal nature of the relationships of the key variables.

Second, this study did not address some influential lifestyle behaviors (e.g., consumption of unhealthy foods, sedentary lifestyle, quality of sleep, and social participation, etc.). It is also recommended to investigate the role of spirituality and religiosity on an individual’s life satisfaction as Thailand is a religious country and Thai people’s health is related to religious beliefs and practices [49]. Addressing these possible sources of statistical variation will provide more insight into the potential for policy and health interventions for older people to improve their life satisfaction [50].

Despite the methodological limitations, the lack of other research in this area means that our findings can be a useful contribution to the study of links between lifestyle behaviors and life satisfaction score among Thai older persons. In addition, the data for this study come from a nationally-representative, population-based sample, and that reduces the bias of reporting false-negative results and increases the robustness of our findings. The reliability and validity of the main endpoint, i.e., life satisfaction, is considered to be accurate because of the application of the SWLS tool which has been widely used in cross-cultural studies. The tool was also pre-tested in the Thai context in this study. The response rate of the study was relatively high, and the sample size was satisfactory for most analyses.

## Conclusions

Our findings have implications for the development of population health policy and social programs which encourage government and health professionals to place more attention and effort into the intrinsic well-being and benefits of healthy lifestyle behaviors for older persons. Life satisfaction among older persons might be positively affected by healthy lifestyle behaviors. In particular, regular physical activity had a strong association with life satisfaction level, while sufficient FV intake had a least association with life satisfaction. Findings also indicate differences in life satisfaction among older persons with sociodemographic characteristics and presence of chronic disease. Collectively, the findings suggest the need for future studies, especially those with longitudinal designs which may provide better information on the direction of the relationship between older persons’ lifestyle behaviors and life satisfaction score. Longitudinal data on other influential lifestyle behaviors (e.g., consumption unhealthy foods, sedentary lifestyle, social participation, etc.) are also needed to identify the key predictors of life satisfaction. Government should pay an attention to promoting healthy lifestyle behavior in older persons by implementing specific interventions, including health and social care services and other support to meet the needs of older persons.

## Supplementary Information

**Additional file 1: Supplementary File 1.** The 2018 Survey Questionnaire on Fruit and Vegetable Consumption among Thai People.

## Data Availability

The date of this study can’t be shared publically due to presence of sensitive (confidential) participants’ information.

## Abbreviations

CI: Confidence Interval
FV: Fruit and Vegetable
ORs: Odds Ratios
SD: Standard Deviation
WHO: World Health Organization
SWLS: Scale with Life Satisfaction
