# Supplementary material for: Influence of healthy lifestyle behaviors on life satisfaction in the aging population of Thailand: a national population-based survey
Source: BMC Public Health. 2021 Jan 6;21:43. doi: 10.1186/s12889-020-10032-9 (PMC7789197; doi:10.1186/s12889-020-10032-9)
Supplement: Supplementary file 1 — Additional file 1: Supplementary File 1. The 2018 Survey Questionnaire on Fruit and Vegetable Consumption among Thai People. [file 12889_2020_10032_MOESM1_ESM.pdf]

"The 2018 Survey Questionnaire on Fruit and Vegetable Consumption among Thai People"

PART 1: GENERAL INFORMATION

1.1: Demographic characteristics

Q1. Sex

- Male
- Female

Q2.1 Birth date ___
Q2.2 Birth month ___
Q2.3 Birth year (BE) ___
Q2.4 Age (year) ___

Q3. Marital status

- Single (including having a partner, but not married)
- Marriage (both registered and non-registered)
- Widow
- Divorce
- Separated

Q4. Religion

- Buddha
- Catholic / Christian
- Protestant / Christian
- Islam
- Brahmin
- Sikkh
- Confucianism / Taoism
- Others (please specify ___)
- No religion

1.2: Socio-economic status

Q5. Highest education level

- Primary school
- Secondary school
- Bachelor
- Higher than bachelor
- No formal education

Q6. Do you currently have a job?

- Yes
- Unemployed / Looking for work (go to Q8)
- Waiting for seasonal job (go to Q8)
- No - work at home (go to Q8)
- No - study (go to Q8)
- No - retired (go to Q8)
- No - unable to work due to sickness or disability (go to Q8)
- No – too old to work (go to Q8)
- Volunteer job (go to Q8)
- Others (please specify ___)

Q7. What is your occupation?

- Government or state enterprise
- Private sector employees
- Business owner
- Laborer
- Farmer
- Others (please specify ___)

PART 2: LIFE SATISFACTION

Q8. Below are five statements that you may agree or disagree with. Using the 1 7 scale below, please indicate your agreement with each item.

- 7 - Strongly agree
- 6 - Agree
- 5 - Slightly agree
- 4 - Neither agree nor disagree
- 3 - Slightly disagree
- 2 - Disagree
- 1 - Strongly disagree

Q8.1 In most ways my life is close to my ideal.
Q8.2 The conditions of my life are excellent.
Q8.3 I am satisfied with my life.
Q8.4 So far I have gotten the important things I want in life.
Q8.5 If I could live my life over, I would change almost nothing.

PART 3: HEALTH STATUS

Q9. Do you wear dentures?

- No
- Yes

Q10. Do you have chronic disease(s)?

- No
- Yes, please specify all the diseases you have.

PART 4: HEALTH RELATED BEHAVIORS

Q11. Have you every smoked in your life (including cigarettes, cigars, pipes, rolling tobacco and baraku)?

- Never
- Yes, but quit already
- Yes, but not regularly
- Yes, with regular smoking

Q12. Have you every drunk alcohol in your life (including whiskey, beer, wine, cocktails, and herbal liquor)

- Never
- Yes, but quit already
- Yes, but not regularly
- Yes, with regular drinking

Q13. Below are six statements that you may agree or disagree with. Please indicate your agreement with each item.

- No
- Yes

Q13.1 Watching TV continuously for more than 1 hour each time
Q13.2 Sitting and chatting / sitting down at a meeting continuously for more than 1 hour each time
Q13.3 Sitting for work or for study continuously for more than 1 hour each time
Q13.4 Sitting to play games or sitting at a computer or smart phone continuously for more than 1 hour each time
Q13.5 Walking-running, biking, indoor cycling, aerobic exercise and/or playing sports at least 30 minutes per day
Q13.6 Doing housework (e.g. washing cloth by hands, gardening, house cleaning, walking around the house) at least 30 minutes per day

PART 5: CONSUMPTION OF FRUITS AND VEGETABLES

Q14. How many days, in the past week, did you eat fruits and vegetables (both cooked and raw)?

- Did not eat
- 1 day / week
- 2 days / week
- 3 days / week
- 4 days / week
- 5 days / week
- 6 days / week
- Everyday

Q15. Below are questions about vegetable consumption. Please indicate frequency and amount of your vegetable consumption in the past week.

Q15.1 How many days, in the past week, do you eat vegetables?

- Did not eat
- 1 day per week
- 2 days per week
- 3 days per week
- 4 days per week
- 5 days per week
- 6 days per week
- Everyday

Q15.2 How many times a day did you normally eat vegetables? _____ time(s)

Q15.3 How many serving(s) of vegetables did you normally eat each time?
_____ serving(s) (number of rice-serving spoons/ladles)

Q15.4 How often, in the past week, did you eat the following groups of vegetables?

Group	Q 15.4.1 Frequency of vegetable consumption (per week)								Q 15.4.2 Number of times a day to eat	Q15.4.3 Average amount of vegetable consumption (each time)	
	None	1 day	2 days	3 days	4 days	5 days	6 days	every day		Sample of one rice-serving spoon/ladle*	Number of serving(s) (rice-serving spoon/ladle)
1. Roots (such as carrot, radish, fingerroot, onion, red onion, ginger, galangal)<br>*not including sweet potatoes										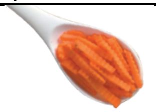<br>1 ladle	
2. Stems and stalks (such as celery, lentils, green/spring onion, garlic chives, morning glory, water mimosa)										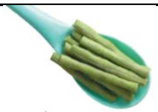<br>1 ladle	
3. Leaves (such as cabbage, kale, spinach, levy gourd, Chinese cabbage (pak-choi, pe-tsai), lettuce, Baegu, Senna siamea, white popinac)										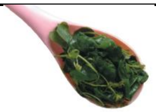<br>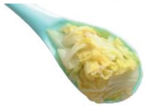<br>1 ladle	
4. Flowers/flower buds with stems/stalks (such as asparagus; broccoli, broccoli raab; Chinese broccoli, cauliflower, green cauliflower, Sesbania grandiflora, Sesbania flower, pumpkin flower, banana blossom, Siamese neem)										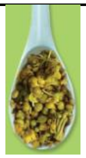<br>1 ladle	

Group	Q 15.4.1 Frequency of vegetable consumption (per week)								Q 15.4.2 Number of times a day to eat	Q15.4.3 Average amount of vegetable consumption (each time)	
	None	1 day	2 days	3 days	4 days	5 days	6 days	every day		Sample of one rice-serving spoon/ladle*	Number of serving(s) (rice-serving spoon/ladle)
5. Fruits (such as cucumber, zucchini, winter melon, green eggplant, eggplant, bitter melon, pumpkin, tomato, twisted cluster bean, sweet/bell - peppers, green/red/yellow)										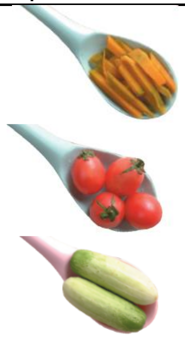 1 ladle	

* Image from Healthy Eating Guidelines (2007) from the Bureau of Nutrition, Department of Health, Ministry of Public Health, Thailand

Q16. Below are questions about fruit consumption. Please indicate frequency and amount of your fruit consumption in the past week.

Q16.1 How many days, in the past week, do you eat fruits?

- Did not eat
- 1 day per week
- 2 days per week
- 3 days per week
- 4 days per week
- 5 days per week
- 6 days per week
- Everyday

Q16.2 How many times a day did you normally eat fruits? _____ time(s)

Q16.3 How many serving(s) of fruits did you normally eat each time? _____ serving(s) (examples of one serving - half a mango, half a Cavendish banana, one cultivated banana, one large tangerine, 6-8 grapes, 4 rambutans, 6 mouth-size pieces of pineapple)

Q16.4 How often, in the past week, did you eat the following groups of fruits?

Group	Q 16.4.1 Frequency of fruit consumption (per week)								Q 16.4.2 Number of times a day to eat	Q16.4.3 Average amount of fruit consumption (each time)	
	None	1 day	2 days	3 days	4 days	5 days	6 days	every day		Sample of one fruit serving*	Number of serving(s) (rice-serving spoon/ladle)
1. Very small-sized fruits (such as grapes, longan, lychee, Jamaican cherry, star gooseberry, Burmese grapes, strawberry, tamarind).										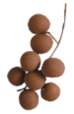 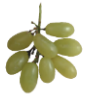	
2. Small-sized fruits (such as rambutan, mangosteen, Marian plum, Sapodilla)										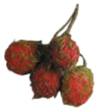 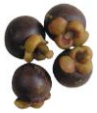	
3. Small-medium-sized fruits (such as cultivated banana, tangerine, apple, pear, star apple)										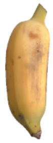 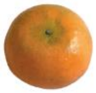	

Group	Q 16.4.1 Frequency of fruit consumption (per week)								Q 16.4.2 Number of times a day to eat	Q16.4.3 Average amount of fruit consumption (each time)	
	None	1 day	2 days	3 days	4 days	5 days	6 days	every day		Sample of one fruit serving*	Number of serving(s) (rice-serving spoon/ladle)
4. Large-medium-sized fruits (such as Cavendish banana, guava, mango, custard apple)										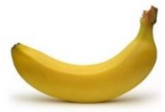 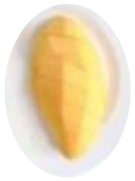	
5. Large-sized fruits (such as watermelon, papaya, pineapple, cantaloupe, coconut)										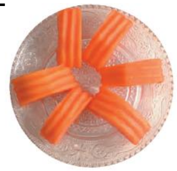 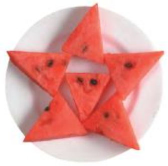	

* Image from Healthy Eating Guidelines (2007) from the Bureau of Nutrition, Department of Health, Ministry of Public Health, Thailand
